# A systematic review of primary care models for non-communicable disease interventions in Sub-Saharan Africa

**DOI:** 10.1186/s12875-017-0613-5

**Published:** 2017-03-23

**Authors:** Jennifer Kane, Megan Landes, Christopher Carroll, Amy Nolen, Sumeet Sodhi

**Affiliations:** 1 Department of Family and Community Medicine, University of Toronto, Toronto, ON Canada; 2 Dignitas International, 2 Adelaide Street West, Suite 200, Toronto, M5H 1L6 Canada; 3 Health Economics and Decision Science (HEDS), School of Health and Related Research (ScHARR), University of Sheffield Regent Court, Regent Street, Sheffield, S1 4DA UK

**Keywords:** Non-communicable diseases, Sub-Saharan Africa, Primary healthcare, Prevention, Treatment, Systematic review

## Abstract

**Background:**

Chronic diseases, primarily cardiovascular disease, respiratory disease, diabetes and cancer, are the leading cause of death and disability worldwide. In sub-Saharan Africa (SSA), where communicable disease prevalence still outweighs that of non-communicable disease (NCDs), rates of NCDs are rapidly rising and evidence for primary healthcare approaches for these emerging NCDs is needed.

**Methods:**

A systematic review and evidence synthesis of primary care approaches for chronic disease in SSA. Quantitative and qualitative primary research studies were included that focused on priority NCDs interventions. The method used was best-fit framework synthesis.

**Results:**

Three conceptual models of care for NCDs in low- and middle-income countries were identified and used to develop an a priori framework for the synthesis. The literature search for relevant primary research studies generated 3759 unique citations of which 12 satisfied the inclusion criteria. Eleven studies were quantitative and one used mixed methods. Three higher-level themes of screening, prevention and management of disease were derived. This synthesis permitted the development of a new evidence-based conceptual model of care for priority NCDs in SSA.

**Conclusions:**

For this review there was a near-consensus that passive rather than active case-finding approaches are suitable in resource-poor settings. Modifying risk factors among existing patients through advice on diet and lifestyle was a common element of healthcare approaches. The priorities for disease management in primary care were identified as: availability of essential diagnostic tools and medications at local primary healthcare clinics and the use of standardized protocols for diagnosis, treatment, monitoring and referral to specialist care.

**Electronic supplementary material:**

The online version of this article (doi:10.1186/s12875-017-0613-5) contains supplementary material, which is available to authorized users.

## Background

Non-communicable diseases (NCDs), primarily cardiovascular disease (CVD), diabetes mellitus (DM), respiratory disease and cancer, are the leading cause of death and disability in the world [1]. In 2010, the Global Burden of Diseases Study estimated global mortality from 235 causes of death and demonstrated evidence of an epidemiological transition from infectious diseases to chronic non-infectious causes of mortality [2]. Underlying this transition in developing countries is a trend towards urbanization, widespread demographic shifts in populations with an increasingly elderly population, and lifestyle changes (such as unhealthy diets and physical inactivity) [3]. In Sub-Sahara Africa (SSA) it is projected that NCD deaths will be greater than communicable, maternal, perinatal and nutritional diseases deaths combined by 2030 [4, 5]. This emerging double burden of disease challenges SSA’s struggling health infrastructure and demands an effective response while recognizing the context of scarce resources within the region [6].

Primary care is for most patients the gateway to the healthcare system, yet in resource-limited settings most primary health care is focused on acute episodic care [7] and chronic disease is often deferred to specialist care delivered at secondary and tertiary centers. Through lessons learned via the implementation of care models for HIV and TB (In the advent of delivering human immunodeficiency virus (HIV) and tuberculosis (TB) care in SSA), we have seen these chronic diseases treated effectively though primary care models for communicable diseases (CD) [8, 9]. Through these primary care models, decentralized clinics for chronic CD care has allowed for rapid and massive scaling up of HIV/TB services, allowing greater access to care primarily for patients in rural locations – by reducing travel time and costs [10]. Currently there is a lack of evidence and guidelines for primary care models focused on the prevention and management of priority NCDs (DM, CVD and respiratory disease) in the SSA context. This systematic review will focus on this knowledge gap and build upon build upon relevant models of disease management in SSA [11]. In this review our aim is to systematically review the literature for evidence to guide the development of primary care models for DM, CVD and respiratory disease.

We have chosen these three disease categories based on the existing literature. The emphasis from the World Health Organization (WHO) and many other steering groups for chronic NCD prevention and management is focused on CVD, DM, cancer and respiratory disease including asthma and chronic obstructive respiratory disease (COPD) [12]. This grouping is based on the common underlying behavioral risk factors of unhealthy diet, physical inactivity, smoking and excess alcohol consumption [4]. We do not focus on cancer diagnosis and management given the increased complexities involved, however we recognize that flexibility should be recognized as a key component of developing an NCD primary care model such that it could respond in future to include other cost-effective interventions such as mental health and cancer integration, which has shown some success in low-cost interventions being piloted and rolled-out [13, 14].

## Methods

With both conceptual primary care models for NCDs in low-and middle-income countries (LMIC) and case studies in SSA of primary care models for NCDs available in the literature, a methodology to synthesize the relevant literature was needed. “Best fit” framework synthesis is a recently described methodology in the literature [15, 16]. It allows a generic conceptual model of care to be built upon via primary research data from a relevant but potentially different population. Best-fit” framework synthesis was the chosen method because it allowed the review to make use of existing primary care models for NCDs, developed for LMICs generally, as the framework against which to code evidence from primary studies specifically conducted in SSA contexts.

First, conceptual models of care for the particular diseases of interest were identified. These models were condensed to key themes to develop an a priori framework. Second, a further systematic review of the literature was undertaken to identify primary research studies focused on primary care interventions for NCDs in SSA, for inclusion in the review. Data from the primary studies are coded based on the a priori themes and any new themes from the primary studies were derived for evidence not included in the a priori framework already. Through the a priori framework and the addition of new themes, the generation of a synthesized new conceptual model of care, relevant to the context of interest, was created through a combination of framework and thematic approaches to synthesis.

### A priori framework

The a priori framework was developed using an approach published by Carroll et al [15]. A systematic search was conducted to identify models and frameworks of primary care interventions in LMICs. Inclusion criteria are outlined in Table 1. A flowchart of the literature search for the a priori framework can be found in Additional file 1.

**Table 1:** Inclusion criteria for the a priori framework models

Setting/Population	Low and middle income countries
Program or intervention focus	Packages of primary care interventions for priority NCDs
Research type/Study design	Publications describing or testing model or framework
Exclusions	•Markov or economic model •Animal model •Model solely focused on health promotion – model of care must include medical management of diseases

Three relevant papers were identified: two applied the directly observed treatment short course (DOTS) model of care approach for TB, previously applied to scaling up HIV care and now adapted for management of NCDs [8, 11, 17]. The remaining model of care adapted the chronic care model (CCM) framework specifically for LMIC to address priority NCDs [18]. Following thematic analysis, a process described elsewhere for developing an a priori framework from more than one relevant model [15] (see also Additional file 2), a framework with 13 distinct themes focusing on diagnosis, prevention and management of priority NCDs was developed from these three models of care. All 13 themes and their definitions are listed in Table 2.

**Table 2:** A priori framework with definitions of themes for coding

Themes derived for coding	Definitions
Case finding	Passive screening for NCDs of patients presenting to local health facilities
Modify risk factors	Everyone seen in primary care should be assessed for common risk factors such as smoking, alcohol, obesity and counseled in lifestyle modifications
Standardized treatment	Algorithm protocol for which medications and dose for DM, asthma, COPD or HTN
Standardized diagnosis	Algorithm outlining protocol for making a diagnosis of DM, asthma, COPD or HTN
Standardized referral pathway	Algorithm with protocol for when to refer a patient needing more complex management to secondary or tertiary care
Standardized follow-up appointments	Guidelines outlining when patients should return for follow-up appointment, ensuring that pre-booked appointments are available at the clinic
Adherence support	Some form of support to patients for adherence to medication and follow up appointments at the clinic (i.e. text message reminder)
Task-shifting/Multidisciplinary clinic	NPC to have the primary role in screening, preventing and managing NCDs
Training of staff	Curriculum to train the health care staff delivering care for NCDs management
Decentralized care	Primary care clinics should be available and accessible to patients living in rural areas
Essential medicines	Consistent supply and access to medicines needed to treat NCDs, primarily drugs outlined in treatment algorithm so that treatment is not interrupted
Essential diagnostics	Essential equipment needed to follow diagnostic protocol for screening and follow-up of NCDs
Systematic monitoring and evaluation	Efficient system for data collection of NCDs (of key indicators such as number died, lost to follow-up, stopped treatment or referral)

*NCD* non-communicable diseases, *COPD* chronic obstructive pulmonary diseases, *HTN* hypertension, *NPC* non-physician clinician

#### Primary research studies

Four electronic database (MEDLINE, Embase, Global Health and CINAHL) were interrogated to identify primary research studies [15] relevant to primary care models for NCDs in SSA. The following MeSH headings were used: non-communicable disease, chronic disease, cardiovascular disease, chronic obstructive lung disease, diabetes, primary care, preventive care and Africa of the South of the Sahara. Using the World Bank listing of countries in SSA, MeSH and key word search was developed to include all individual countries. An example of the full search strategy can be seen in Additional file 3. A grey literature search was conducted of Google Scholar, African Wide Info and Eldis using the same combination of key terms. Bibliographies of key articles were also searched for additional references. This iterative approach helped cast the net further to draw on relevant studies focused on primary health care approaches for the management of NCDs in SSA.

The inclusion criteria applied to primary research studies from the literature search are described in Table 3. Given the focus of this synthesis on models of care for NCD management, studies looking at both quantitative and qualitative outcomes were included, allowing an exploration of intervention descriptions and how the different models of care work in context in SSA. The synthesis is descriptive in nature and does not link models of care to intervention success.

**Table 3:** Inclusion criteria for the primary research studies

Setting/Population	Sub-Saharan Africa
Program or Intervention Focus	Packages of primary care interventions for priority NCDs
Research type /Study design	Quantitative and qualitative research studies describing intervention or development of package of care
Exclusions	•Interventions solely base on health promotion – intervention must include medical management of diseases •Children/pediatric •Not focused on 1 of 3 priority NCDs management (CVD, asthma/COPD, DM)

*NCD* non-communicable disease, *CVD* cardiovascular disease, *COPD* chronic obstructive pulmonary disease, *DM* diabetes mellitus

#### Data extraction

A data extraction form for the primary research studies was developed that focused on study design, location, type of NCD, intervention and outcomes, along with the themes outlined in the a priori framework. The evidence coded against the thematic framework came from the description of the intervention itself, and the quantitative and qualitative outcomes in the results section of the paper. Two authors (JK, AN) independently extracted data from the studies using this deductive approach. The synthesis also involved the secondary thematic analysis of evidence not captured by the themes of the a priori framework, This inductive approach allowed the authors (JK, AN) to add to the known themes of the framework and thus to develop a the thematic framework specifically for NCDs in SSA.

#### Consideration of study quality

Two authors (JK, AN) undertook independent quality assessment of the included studies using tools adapted for critically appraising both qualitative, quantitative and mixed method studies [19, 20]. No sensitivity analysis based on study quality was performed because none of the studies was found to be of poor quality: the quality of the included studies was generally similar.

#### Generation of a new model of care framework

Following the systematic review, the authors (JK, AN) reviewed again the a priori concepts and the list of new themes generated from the primary studies. This resulted in a finalized list key to the model of care for NCDs in SSA. Revisions were also made to some of the a priori themes as a result of the synthesis. The synthesis then involved looking for relationships and common characteristics between themes to derive higher-level concepts. This allowed the analysis of the a priori framework and primary data to move beyond simply a description of a list of themes to a synthesized conceptual model of care for NCDs in SSA. A new finalized model of care from this synthesis was presented.

## Results

### Quantity and quality of included studies

**Fig. 1:**
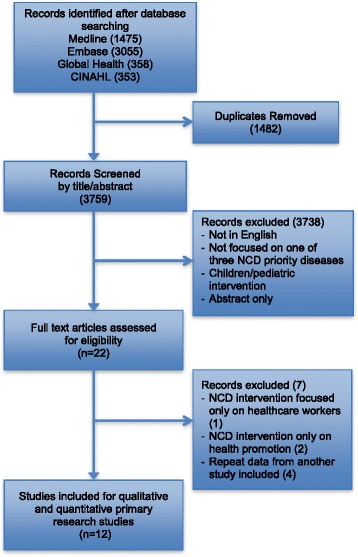
PRISMA flowchart detailing results of literature search and study screening of Primary Research Studies

The search generated 3759 unique citations from across four databases. Twenty-two full papers from theses results were screened of which 12 satisfied the inclusion criteria (Fig. 1). Results from the grey literature and bibliographic references of key articles did not generate any additional studies relevant to the selection criteria. All studies were appraised for quality using the relevant methodology [19, 20] and none was assessed as low quality. The results are summarized in Additional file 4.

A summary of the features of the primary research studies included is included in Table 4. The 12 primary research studies were conducted in Kenya [21, 22], Ethiopia [23, 24], Uganda [25], South Africa [26–28], Nigeria [29], Cameroon [30, 31] and Tanzania [32]. Eleven of these studies were quantitative [21–26, 28–33] one was mixed methods [27] and none was exclusively qualitative in methodology. Two of these studies were early in the development of the intervention and had limited evaluative component [21, 24]. Seven of the studies focused on care delivered solely in a rural setting [21, 22, 24–26, 28, 30] while two focused exclusively on urban primary care of services [23, 32]. Three of the studies included care delivery in both urban and rural settings where the package of care was delivered [27, 29, 31]. As per the inclusion criteria all of the interventions included a package of care for at least one of the priority NCDs - DM, CVD and asthma/COPD. Three of the studies focused on DM care alone [23, 24, 26] and two focused on hypertension (HTN) alone [29, 32]. Four of the studies focused on HTN and DM management together [22, 25, 27, 30] and two studies developed an intervention focused on HTN, DM and asthma together [28, 31]. None of the interventions looked at a package of care for COPD.

**Table 4:** Description of primary research studies

Article	NCD Focus	Study Design	Location	Intervention	Outcome	Quality^a^
Pastakia 2013	DM & HTN	Feasibility study	Rural Kenya	Community vs. home based screening	Low follow up at health center, HTN 31%, DM 22–23% follow within 3 months	Adequate
Rabkin. 2012	DM	Pre/post intervention	Urban Ethiopia	Protocol of DM care implemented for HIV patients	Increase BP measurements, fundoscopy exams, booked next appointment after intervention	Adequate
Chamie 2011	DM & HTN	Feasibility study	Rural Uganda	POC testing for NCD screening alongside HIV testing campaign	Moderate follow up at health center, HTN 43% and DM 61% a health center	Adequate
Price 2011	DM	Observational cohort study	Rural South Africa	Empowerment based education about DM, clinical algorithm	Hba1c at baseline 10.8, decreased to 7.5 at 18 months, 9.7 at 4 years	Adequate
Bloomfield 2013	CVD & Pulmonary	Program description	Rural Kenya	Twining relationship for academic model for NCD clinical care	No evaluation phase yet, description of model for academic partnership	Adequate
Mendis 2010	HTN	Cluster Randomized trial	Urban/rural Nigeria	WHO CVD risk management package vs. standard care for HTN	SBP and DBP were lower in Nigerian group (p = 0.0002), 2% of patients referred to next level of care, decreased BMI, smoking, increased fruits and vegetables	Adequate
Labhart 2010	HTN & DM	Observational cohort study	Rural Cameroon	Implementation of package of care for HTN/DM for NPC (75 clinics)	Retention of patients at 1 year 18.1%, SBP decreased 22.8 mmHg/DBP decreased 12.4 mmHg/FPG decreased by 3.4 mmol/L (*p* < 0.001)	Adequate
Kengne 2009	HTN, DM & asthma	Feasibility study	Urban/rural Cameroon	Implementation of package of care for HTN/DM/asthma at PHC (5 clinics)	Decrease of SBP 11.7 and DBP 7.8 (*p* < 0.001), decrease 1.6 mmol/L (*p* < 0.001), decreased days with asthma attacks in follow up at 2 years	Adequate
Katz 2009	DM & HTN	Observational cohort study	Urban/rural South Africa	Chronic care model clinic for DM and HTN implemented	Half lost to follow up (49%), 55% of DM patient referred to specialist clinic (76% of these didn’t need referral), 31% of DM controlled with hba1c <7%	Adequate
Bovet 2008	HTN	Prospective population based survey	Urban Tanzania	Health care services after positive screening test for HTN	34% sought health-care provider in 12-mth period, anti-HTN taken by 34% at some point, 3% at end of 12 month follow-up	Adequate
Mamo 2007	DM	Program description	Rural Ethiopia	Implementation of RN-led decentralized NCD clinics	75% of DM patients attended FU appointments, only 11.4% of DM patients could be transferred to PHC clinics because lack of insulin supply at PHC	Adequate
Coleman 1998	HTN, DM & Asthma	Observational cohort study	Rural South Africa	Implementation of RN-led NCD package of care intervention	RN’s able to control 68% of HTN, 82% of DM (NIDDM), 84% of those with asthma	Adequate

^a^If a paper had >50% of the CASP and MMAT checklist then the study was deemed of adequate quality assessment

### Creation of a new conceptual framework

The data from these primary studies was used to help construct an evidence-based conceptual model of care for management of NCDs in SSA. The majority of the themes identified in the a priori model could be identified in and were supported by the primary data for this review. The results of this coding against the a priori concepts can be seen in Tables 5 and 6 (the full details of each theme in each study are provided in Supplementary material, Additional files 5 and 6).

**Table 5:**
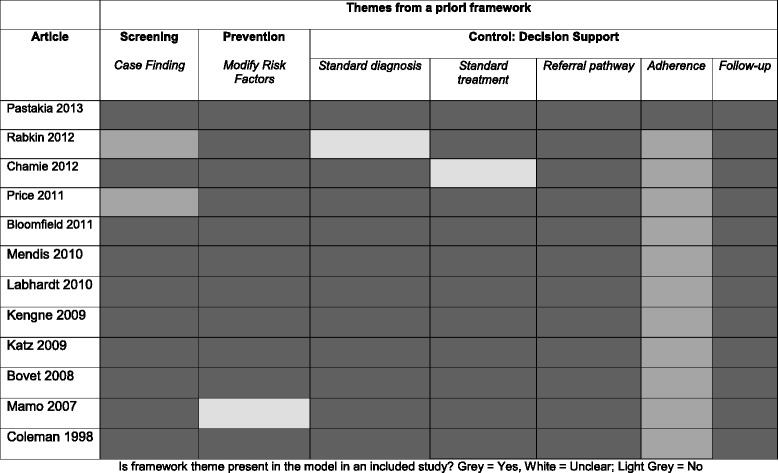
A Priori themes in primary research studies for NCDs interventions in SSA

Is framework theme present in the model in an included study? Grey = Yes, White = Unclear; Light Grey = No

**Table 6:**
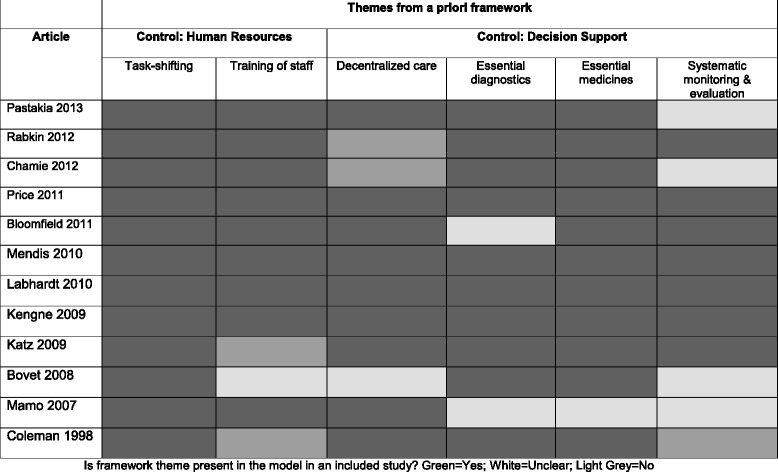
A Priori themes in primary research studies for NCDs interventions in SSA

Is framework theme present in the model in an included study? Green = Yes; White = Unclear; Light Grey = No

### The new conceptual model of care

In deriving the new conceptual model of care, the a priori theme of “training of staff” was slightly altered to “train and retrain staff” as this was more consistent with what was found in the primary studies. Although there was minimal data in the primary studies relevant to the theme of adherence, it was further revised to “adherence to medications” and “adherence to follow-up appointments” to echo the interventions and results in the data.

There were four new themes from the primary studies looking at NCD interventions in SSA. These themes were: “staff competence”, “dedicated NCD staff”, “review criteria” and “communication with MD/specialist”. The a priori model failed to capture these themes, but because the included studies were principally conducted in rural settings in SSA, these new themes are relevant to the context and setting of implementing a model of care in SSA.

These revisions and additions to the a priori model helped to create, through a deductive and inductive approach, a synthesized evidence-based NCD model of care for SSA that can be seen in Table 7.

**Table 7:** A priori including the new concepts and themes

A priori concepts	New concepts	A priori themes	Revised and new themes
Screening		Case finding	
Prevention		Modify risk factors	
Control	Quality improvement		Review criteria
			Staff competence
	Health systems	Essential medicines	
		Essential diagnostics	
		Systematic monitoring and evaluation	
		Decentralized care	
	Decision support	Standardized treatment	Adherence to medications
		Standardized diagnosis	Adherence to follow-up
		Standardized referral pathway	Communication with MD/specialist
		Standardized follow-up appointments	
	Human Resources	Task-shifting/Multidisciplinary clinic	Train and retrain staff
			Dedicated NCD staff

The new conceptual model of care is centered on three higher concepts: screening, prevention and control of NCDs. The majority of existing interventions found in this review fall within the control concept, which can be further broken down into four categories: quality improvement, human resources, decision support and health systems. These themes are further illustrated with evidence from the primary data.

### Screening

The articles describing this element clearly articulate that screening of individuals with NCDs should take a *passive case finding* approach by opportunistically screening patients presenting to health centers. The authors of these models advocate not taking an active case finding approach as its felt to be too labor intensive in a resource poor setting. Eight of the interventions did have a screening component to the them [21, 22, 25, 27, 29–32], however three studies were discordant with this a priori theme by embracing an active case finding approach, either going door-to door or holding community fairs [22, 25, 32]. The common measured outcome of these studies was a very poor linkage to care for those identified to screen positive for the priority NCDs. Although the concept of active case finding can seem ideal to reach more individuals, in context these primary studies reinforce that passive case finding is a more cost-effective approach in SSA.

### Prevention

All of the primary research studies described an element of *modifying risk factors* for NCDs through counseling of patients. The various topics for counseling included: dietary advice, physical activity, obesity, salt intake and smoking cessation. One study by Price et al. [26] reported on the impact of empowerment by education around risk factors and disease progression delivered monthly over the first 3 months of care. Education alone in this study showed a significant decrease in HbA1c at 18 months from 10.6 +/- 4.2% to 7.6 +/- 2.3% (*p* < 0.001).

### Control

#### Quality improvement

Two of the primary studies incorporate *review criteria* of a minimum standard of care for primary care clinicians as an important element of a package of care for NCDs [27, 31]. Maher et al. [11] stresses in his conceptual framework for NCDs in LMIC that care must attain a minimal standard of quality to have improved patient outcomes and Kengne et al. [31] describe review criteria as “must do” criteria for clinics delivering the NCD package of interventions to ensure quality standards. For diabetes care delivered in Soweto, authors incorporated criterion-related validity of appropriate referrals to specialist [27]. This study demonstrated that very few of the patients referred to specialist care in fact needed to be referred (<20%); these patients instead could have been treated at the primary care level. This process of quality standards through review criteria ensures an intervention is implemented successfully and effectively for patients and the limited health care resources.

Similarly in ensuring quality of care, *competency* amongst health care workers must be included in a package of care for NCDs. Four of the primary studies mentioned protocols to ensure clinical competency amongst mainly non-physician clinicians (NPC) [22, 27, 30, 31]. This included pre and post questionnaires after training, observed patient encounters by senior staff and key informant interviews of physicians on NPC performance. Katz et al. [27] demonstrated through questionnaires that trained RN’s for NCD care were more knowledgeable than untrained however only 31% were familiar with the current DM guidelines. Further qualitative data from this study showed physicians working with the RN’s expressed concern of their lack of knowledge of targets to control DM or HTN. This evaluation of competency allowed the clinics to realize gaps in clinical knowledge amongst NPCs and refine teaching on certain topics to ensure a competent trained primary health care (PHC) team delivered care.

#### Health systems

Most of the primary studies for this review had *essential medications* available at the PHC [21, 23, 25–31]. At a minimum, a first line medication was available. Essential medications available at the local primary health care clinic is vital to controlling NCDs. This theme was made clear in the a priori and primary research articles, referring to lessons learned from ART for the HIV epidemic and the DOTS program for TB and how important uninterrupted access to medications for chronic disease control is. *Essential diagnostics* included a glucometer and a blood pressure machine available for all the interventions, as most focused on DM and HTN. *Systematic monitoring and evaluation* as described in the a priori model is a means of collecting data on patients in NCD clinics. Six interventions had a standard checklist or flow sheet that they collected patient’s information on [21, 23, 27, 29–31], none of these were electronic health records. *Decentralized* primary health care for NCDs in SSA allows individuals living in rural and remote locations access care [34]. This concept was incorporated into all but two of the studies, looking at rural locations for implementation of their clinics [21, 22, 24–28, 30, 31].

#### Human resources

*Task shifting* has been an essential component of HIV care delivery in SSA [35, 36] and likewise will be a necessity to how NCD care is delivered in SSA given the health work force shortage. Task shifting gives clinical responsibilities typically done by physicians to less specialized health care workers to deliver care more effectively in resource-limited settings [36]. All of the primary studies had some element of task with eight of the primary research studies describing NPC led clinics, predominantly by RN’s [21, 24, 26–31, 37].

Providing training for staff is indispensable when a large part of the workforce is transitioning to new roles and responsibilities and the theme of *train and retrain* is an important element to several of the interventions described in the primary literature [23, 24, 29–31]. This ongoing training allows, in this resource challenged health care system with high staff turnover, the ability of new staff to be educated and deliver NCD management on an ongoing basis. Katz el al [27]. described a mean staff turnover of 32 +/- 24% per clinic in South Africa, with some clinics as high as 75%. Retraining incorporated into interventions such as in Labhardt et al. [30] allowed staff already trained a refresher course on NCDs.

Integrating NCD clinics within CD can have several advantages for chronic disease management. As seen by the a priori framework, the models of care for NCD management in LMIC embrace the public health approach adapted for communicable disease control such as HIV and TB [8, 11, 17]. That integration however needs to be balanced with the NPC workload. What would seem as ideal, to have one NPC trained for HIV, TB and NCD primary care, can stretch RNs or health care workers. This theme was interpreted as *dedicated NCD staff*, meaning not simply asking staff to do more roles but either hiring more staff for NCD care or giving dedicated time to NPC already working in clinics to manage increase case load of NCD patients [27].

#### Decision support

*Standardization of diagnosis* and *treatment* help ensure quality of standards for clinical care. Ten of the studies had a standardized diagnostic algorithm to follow [21, 22, 25–32]. Treatment algorithms were also included in seven of the studies [21, 23, 26–30], with three studies providing step-by-step protocols for medication [28, 30, 31].

*Standardized referrals* ensure that primary care clinics have a specialist available for patient’s management of disease when it is beyond their scope. Most studies supported when patients were on maximum therapy and their NCD was still not controlled would be referred to secondary or tertiary clinics [24, 27, 28, 30, 31]. Other standard reasons for referral were significant comorbidities (cardiovascular disease, chronic kidney failure) or requiring insulin [26, 27, 29].

As resource limited settings embrace task shifting, ease of access to consult physicians was an important theme to be integrated into the model of care. This theme was categorized as *communication with MD/specialist*. Having a referral pathway was indispensable but more immediate communication with a doctor or specialist could optimize patient management [27]. Two expressed examples of this from an RN led NCD intervention:

*“No doctors help with up-scaling of medications (and) nurses hands (are) tied behind their back as they have responsibility without authority.”* [27]

*“We need to be able to communicate with specialists easily in case of problems we encounter about patients management.”* [27]

Beaglehole et al. [18] point out in their conceptual primary care model that *standardized follow-up appointments* are needed for patients with chronic diseases. Health care predominantly lacks structure in SSA and tends to be focused on acute episodic care, without any planned follow up. This primary health care system redesign requires a different organizational structure in place. The vast majority of the studies had scheduled appointments but a more significant theme was lack of compliance with these scheduled follow-up appointments. Although none of the studies had implemented any reminders for *adherence to follow-up appointments*, this theme was interpreted as being very important given the high attrition rates across the studies. Labhardt et al. [30] retained 18% of patients at one year in their study and Katz et al. [27] lost 49% of NCD patients from their clinics by two years. Price et al. [26] started with 320 patients in their cohort, ending up with 80 at the end of four years in their study. Further planning needs to be centered on this important concept.

There was very little study data to code against the theme of *adherence to medications*. More research is needed to understand if adherence is a problem and if so, how to promote medication adherence amongst NCD patients. Coleman et al. [28] give some thought to this in their study as they had patients return to clinic for a monthly visit until their disease was controlled. Then they allowed a bi-annual clinic visit for their hypertension, diabetes or asthma that were well controlled. This allowed patients to be prescribed medication for 6 months without a clinical review. Overall their adherence to medication (self-reported) was 87%.

## Discussion

By identifying several conceptual models for packages of care for NCDs and combining evidence from primary research studies, this review used a “best fit” framework synthesis to develop a new conceptual model of care for NCDs in SSA. This review aimed to assess what features characterize models of primary care for NCDs in SSA by focusing on the interventions themselves and the mechanisms behind these interventions. This was not with the goal of determining the most effective model of care. In identifying models of care for NCD in LMIC for the a priori framework, two of the three frameworks were already focused on SSA. Data from the primary research studies supplemented the a priori themes but likely less so than if these models were more generic for all LMICs.

The a priori themes emphasized an overall change in approach to NCDs in LMICs, moving from acute, episodic care focused on individuals to a public health programmatic structure focused on organized monitoring and evaluation of patient outcomes, standardized quality care and systematic follow up [8, 11, 17, 18]. Adapting disease guidelines in the face of extreme shortages of health care workers requires NPC to be delivering care [34] and to ensure effective implementation standardized protocols for diagnosis, treatment and monitoring need to be in place [38]. This simplification of management protocols builds upon the success of HIV service delivery in resource poor settings as demonstrated by the DOTs framework [8, 39]. Furthermore, this standardized public health approach to disease control goes beyond the individual clinic level and can inform population based interventions. For example, routine data collection via standardized protocols for NCDs at each clinic can inform on the national burden of disease and further contribute to accurate drug forecasting [8].

Further, this public health approach stresses opportunistic case finding as the most cost-effective intervention for screening in SSA [8, 11, 17, 18]. This was re-emphasized in the primary studies demonstrating very poor linkage to care for active case finding [22, 25, 32]. However Beaglehole et al. point out some obstacles when opportunistic case finding is operationalized [18]. Patients will often be presenting with acute issues and providers will need to be proactive screening for asymptomatic NCDs within a limited consultation time. This may need further consideration and adaptation with more operational research in SSA.

New themes derived from the primary studies emphasize contextual factors unique to SSA. Health worker shortages are prevalent across all LMIC however it is the extreme case in SSA. The ratio of physician to patient is as low as 1 per 1000 in Tanzania and 2 per 1000 in Malawi [40]. As a result with the majority of care delivered via NPCs in SSA there needs to be appropriate quality assurance. Ensuring quality of care was mentioned in the conceptual models of care however specifics of how to operationalize this were more vague. The concept of quality improvement including both staff competence and review criteria were made clear through the operational primary research studies in SSA. This likely reflected that with NPCs being crucial to scaling up care in SSA there needs to be more regulation to address greater variability in the care delivered.

Aside from increasing the sheer number of NPCs via additional training and funding, incentives and creating a better work environment to retain health care workers is essential [18]. The new themes derived from primary research in SSA of communication with MD/specialist and dedicated NCD staff emphasizes this point. Given increasing responsibility, NPCs need more access to senior health care workers to manage patients and with an already large burden of workload with communicable disease management adding further patient load caring for NCDs will need to be done cautiously.

The emphasis of this review was to use the literature to develop a medical model of care for NCDs in SSA, focused specifically on CVD, DM, and respiratory diseases – chronic obstructive pulmonary disease (COPD) and asthma. This excluded studies solely focused on health promotion, education or other psychosocial interventions. Although the non-medical management of NCDs is essential to a package of care, this review aimed to summarize a model of care that could be implemented at decentralized primary care clinics in SSA.

How exactly this model of care would be implemented gives way to the Alma Ata discussion of selective versus comprehensive health care [34]. HIV care has typically been implemented as a vertical program in LMIC. The infrastructure already developed in SSA for the delivery of chronic care for HIV, could be leveraged for an integrated horizontal program for NCDs [9, 41–43]. A case example in Swaziland of an NCD clinic for DM and HTN not having booked appointments, standardize treatment protocol, referral system and patient counseling despite being in the same clinic delivering HIV care having these tools and strategies demonstrates missed opportunity of leveraging an already present infrastructure [44]. With an increasing number of individuals on ART in SSA, the increased risk of diabetes and hyperlipidemia associated with ART suggests potential synergies in a horizontally integrated HIV-NCD model of care [45].

Finally, it is worthy of note that this is the first instance of “best fit” framework synthesis being conducted on data from quantitative and mixed method studies [15, 16]. All previous published examples of the method have focused on the synthesis of qualitative evidence alone [15, 16]. This evidence synthesis has demonstrated that “best fit” is equally viable as an approach for synthesizing quantitative and/or mixed method studies: the method facilitates the identification and development of a relevant a priori framework, against which quantitative data can be coded equally as well as qualitative data. Given the relative lack of detailed guidance on how to conduct narrative synthesis in systematic reviews [46], “best fit” framework synthesis clearly offers a well-specified approach that can make sense of studies and evidence that cannot be readily combined using statistical or qualitative approaches.

### Limitations of this review

The focus of this review was on SSA, to help build a relevant contextual model of care for this area. Of the forty-eight countries in SSA, only seven SSA countries had primary studies that fit the inclusion criteria. Although limited in numbers of countries that had operational data, the consistent themes derived from these studies suggest these could likely be generalizable to other parts of SSA.

Many different study designs were included in this review, looking at different outcomes (primary and secondary) and different interventions. It had been explored doing a more quantitative synthesis of certain outcomes such as Hba1c or BP improvement with packages of care in SSA, however the interventions and outcomes were too heterogeneous in the studies to summarize them in such a way. Through secondary thematic analysis common themes stemmed from the literature, however it is more difficult given the diversity of study design and focus to make generalizations.

Another limitation of the quantitative studies, predominantly cohorts, was the high attrition rate. Although the outcomes and results were used informally to feedback about the interventions contextual factors to build a model of care, the low retention rates of patients in the studies speaks to the interventions themselves and risk of bias in the outcomes. These studies need to be interpreted with caution.

## Conclusions

Despite the limitations of the data, this review conducted a thematic analysis to develop a conceptual model of care for NCDs in SSA. This medical model of care emphasized three major concepts for the components of an intervention; screening, prevention and control. Furthermore, following synthesis, the control of NCD component could be further broken down into: health systems, quality improvement, decision support and human resources, each with more detailed themes that contextualized these concepts. The scarcity of data available for this important research topic emphasized the urgent need for more research. Specifically further research looking at the effectiveness of these models of care is essential to help roll out piloted research to then scale up for national programs. Political will and commitment must be combined with increasing public awareness to harness resources and global attention for addressing this growing epidemic of NCDs in SSA.

## Additional files

Additional file 1: Literature results for the a priori framework. (DOCX 25 kb)

Additional file 2: Models of care for NCDs in LMIC. (DOCX 16 kb)

Additional file 3: Example of search terms for primary research studies. (DOCX 16 kb)

Additional file 4: Quality assessment of primary research studies. (DOCX 97 kb)

Additional file 5: A Priori themes in primary research studies for NCDs Interventions in SSA. (DOCX 18 kb)

Additional file 6: A Priori themes in primary research studies for NCD interventions in SSA. (DOCX 17 kb)

## Abbreviations

CD: Communicable disease
COPD: Chronic Obstructive Pulmonary disease
CVD: Cardiovascular disease
DM: Diabetes mellitus
HTN: Hypertension
LMIC: Low-and middle-income countries
NCD: Non-communicable disease
NPC: Non-physician clinician
PHC: Primary health care
SSA: Sub-Saharan Africa
