# Supplementary material for: A systematic review of primary care models for non-communicable disease interventions in Sub-Saharan Africa
Source: BMC Fam Pract. 2017 Mar 23;18:46. doi: 10.1186/s12875-017-0613-5 (PMC5363051; doi:10.1186/s12875-017-0613-5)
Supplement: Supplementary file 1 — Literature results for the a priori framework. (DOCX 25 kb) [file 12875_2017_613_MOESM1_ESM.docx]

Records identified after database searching

Medline (1969)

Embase (3809)

Global Health (326)

Records Screened

by title/abstract

(5089)

Duplicates Removed

(1015)

Records excluded (5084)

- Not in English
- Abstract only
- Not LMIC setting
- Not theory/model of care
- Not priority NCD focus (CVD, DM, respiratory)

Full text articles assessed

for eligibility

(n=5)

Studies included to develop an *A Priori* Framework

(n=3)

Records excluded (2)

- Repeat theory/model from another study included

Additional file 1: Literature Results for the *A Priori* Framework
