# Supplementary material for: A systematic review of primary care models for non-communicable disease interventions in Sub-Saharan Africa
Source: BMC Fam Pract. 2017 Mar 23;18:46. doi: 10.1186/s12875-017-0613-5 (PMC5363051; doi:10.1186/s12875-017-0613-5)
Supplement: Supplementary file 2 — Models of care for NCDs in LMIC. (DOCX 16 kb) [file 12875_2017_613_MOESM2_ESM.docx]

| **Maher et al. 2010, 2009**  *“Health Transitions in Africa: Practical Proposals for primary care”, “A global framework for action to improve primary care response to chronic non-communicable diseases: a solution to a neglected problem”*  Adapted DOTS Framework | **Beaglehole et al. 2008**  *“Improving the prevention and management of chronic disease in low-income and middle-income countries: a priority for primary health care”*  Adapted CCM framework | **Harries et al. 2008**  *“Adapting the DOTS Framework for Tuberculosis Control to the Management of Non-communicable Diseases in Sub-Sahara Africa”*  Adapted DOTS framework | **Themes** |
| --- | --- | --- | --- |
| Case finding among patients presenting to local health facilities, screen for common NCDs, low-cost, reliable and rapid means | Opportunistic case finding, detection of early disease | Passive case finding (those presenting to health facilities), active case finding is too labor intensive | Case finding |
| Identify and address modifiable risk factors for everyone seen in PHCs (smoking, alcohol, obesity, lifestyle modification), standardize protocol for health promotion | Assessment of risk factors, identify high risk individuals | Lifestyle modification essential part of this endeavor (as HIV prevention essential to ART delivery) such as diet, exercise, smoking, alcohol for prevention of NCDs | Modify risk factors |
| Simple standardized treatment protocol | Decision support through development of guidelines | Simple treatment protocol, not DOT but self-administered as life-long | Standardized treatment |
| Standard diagnostic protocol for NCD case-finding | Decision support through development of guidelines | Simple diagnostic protocol | Standardized diagnostics |
| Standardized protocol when needed for referrals, Framework with referral of patients with NCDs needing more complex management to secondary or tertiary care | Continuous access to professionals with expertise in experience of specific illnesses | Active tracing of patients who have been referred elsewhere | Standardized referral pathway |
| Standardized protocol for follow-up | Planned follow-up visits | Active tracing of patients who fail to attend clinics | Standardized follow-up appointments |
| Long-term adherence support | Improvement of adherence to treatment |  | Adherence support |
| Multidisciplinary chronic disease clinics | Non-physician clinicians will have a leading role in preventing/managing chronic disease |  | Task-shifting / multidisciplinary clinic |
| Train primary care staff to be able to deliver the strategy for primary care response | Train and retain a health workforce, ensuring right clinical competencies |  | Training of staff |
|  | Provision has been focused on hospital and clinics, a need to extend provision to rural settings in which facilities are lacking |  | Decentralized care |
| Improving quality of clinical care by reliable drug supply | Robust supply system for drugs | Access to essential medicines, monitor to ensure accurate drug forecasting | Essential medicine |
| Well equipped health care environment with good diagnostic support Essential materials for diagnosis, simple and inexpensive tools | Robust supply for equipment | Access to essential diagnostic equipment for following diagnostic protocol | Essential diagnostics |
| Data collection system for standardized monitoring & evaluation of outcomes, efficient system for data collection, patient held record, record keeping for health facilities, key indicators | Use of clinical information systems to monitor patients clinical health outcomes | Treatment master cards for specific NCDs to provide up to date case burden and treatment outcomes | Systematic monitoring & evaluation |

Additional file 2: Models of Care for NCDs in LMIC *(DOTS = Directly observed treatment short course, CCM = Chronic care model, NCDs = Non-communicable diseases, PHC = Primary health care, ART = Antiretroviral therapy)*
