# Supplementary material for: A systematic review of primary care models for non-communicable disease interventions in Sub-Saharan Africa
Source: BMC Fam Pract. 2017 Mar 23;18:46. doi: 10.1186/s12875-017-0613-5 (PMC5363051; doi:10.1186/s12875-017-0613-5)
Supplement: Supplementary file 3 — Example of search terms for primary research studies. (DOCX 16 kb) [file 12875_2017_613_MOESM3_ESM.docx]

Additional file 3: Example of Search Terms for Primary Research Studies

Embase Database

| 1. exp non communicable disease or exp chronic disease or exp diabetes mellitus or exp cardiovascular disease or exp blood pressure or exp hypertension or exp coronary artery disease or exp cholesterol or exp chronic obstructive lung disease or exp chronic bronchitis or exp emphysema or exp asthma | 4,160,032 |
| --- | --- |
| 2. chronic* disease* or NCD* or noncommunicable* disease* or non-communicable* disease* or non* communicable* disease* or diabete* or cardiovascular* disease* or hypertension* or blood* pressure* or cholesterol* or coronary* artery* disease* or COPD* or chronic* obstructive* pulmonary* disease* or asthma* or chronic* bronchitis* or emphysema* | 2,377,194 |
| 3. 1 or 2 | 4,437,776 |
| 4. exp primary medical care or exp preventive medicine or exp health care delivery | 1,892,263 |
| 5. primary* health* care* or prevent* medicine* or prevention* or primary* health* service* or primary* medical* care* or package* of* care* | 797,798 |
| 6. 4 or 5 | 2,493,591 |
| 7. exp "Africa south of the Sahara" or exp South Africa or exp Central Africa or Angola or Atlantic Islands or Benin or Botswana or Burkina Faso or Burundi or Central African Republic or Cameroon or Cape Verde or Chad or Comoros or Congo or Cote d’Ivoire or “Democratic Republic of the Congo” or Eritrea or Ethiopia or Gambia or Gabon or Ghana or Guinea or Guinea-Bissau or Kenya or Lesotho or Liberia or Madagascar or Malawi or Mali or Mauritania or Mauritius or Mozambique or Namibia or Niger or Nigeria or Rwanda or Senegal or Seychelles or Sierra Leone or Somalia or South Africa or Sudan or Swaziland or Tanzania or Togo or Uganda or Zambia or Zimbabwe | 161,120 |
| 8. sub* sahara* africa* or sub-sahara* africa* or south* africa* or Angola or (Benin or Dahomey) or (Botswana or Bechuanaland or Kalahari) or (Burkina Faso or Burkina Fasso or Upper Volta) or Burundi or Cape Verde or (Central African Republic or Ubangi-Shari) or Chad or (Congo not ((democratic republic adj3 congo)) or (Cote d’Ivoire or Ivory Coast) or (Comoros or Comoro Islands or Mayotte or Iles Comores) or ((democratic republic adj2 congo) or Belgian Congo or Zaire) or Eritrea or Ethiopia or (Gabon or Gabonese Republic) or Gambia or ((Guinea not (New Guinea or Guinea Pig* or Guinea Fowl)) or (Ghana or Gold Coast) or (Guinea Bissau or Portuguese Guinea) or Kenya or (Lesotho or Basutoland) or Liberia or (Madagascar or Malagasy Republic) or (Malawi or Nyasaland) or Mauritania or (Mauritius or Agelega Islands) or (Mozambique or Portuguese East Africa) or Namibia or ((Niger not (Aspergillus or Peptococcus or Schizothorax or Cruciferae or Gobius or Lasius or Agelastes or Melanosuchus or radish or Parastromateus or Orius or Apergillus or Parastromateus or Stomoxys)) or Nigeria or (Rwanda or Ruanda) or (Sao Tome adj2 principle) or Senegal or Seychelles or South Africa or Sierra Leone or Sudan or Swaziland or Somalia or (Tanzania or Zanzibar) or (Togo or Togolese Republic) or Uganda or (Zambia or Northern Rhodesia) or (Zimbabwe or Rhodesia) | 205,394 |
| 9. 7 or 8 | 208,760 |
| 10. 3 and 6 and 9 | 3,055 |
