# Supplementary material for: A systematic review of primary care models for non-communicable disease interventions in Sub-Saharan Africa
Source: BMC Fam Pract. 2017 Mar 23;18:46. doi: 10.1186/s12875-017-0613-5 (PMC5363051; doi:10.1186/s12875-017-0613-5)
Supplement: Supplementary file 4 — Quality assessment of primary research studies. (DOCX 97 kb) [file 12875_2017_613_MOESM4_ESM.docx]

Additional file 4: Quality Assessment of Primary Research Studies

Table A - Quantitative Studies Quality Appraisal (green=yes, yellow=unknown, red=no)

| **Study** | **Focused issue?** | **Cohort recruitment?** | **Exposure measurement?** | **Outcome measurement?** | **Confounding Factors?** | **Follow-up of subjects?** | **Result of study?** | **Applicable to local population?** |
| --- | --- | --- | --- | --- | --- | --- | --- | --- |
| Pastakia 2013 |  |  |  |  |  |  |  |  |
| Chamie 2012 |  |  |  |  |  |  |  |  |
| Bovet 2008 |  |  |  |  |  |  |  |  |
| Rabkin 2012 |  |  |  |  |  |  |  |  |
| Labhardt 2010 |  |  |  |  |  |  |  |  |
| Price 2011 |  |  |  |  |  |  |  |  |
| Coleman 1998 |  |  |  |  |  |  |  |  |
| Kengne 2009 |  |  |  |  |  |  |  |  |
| Bloomfield 2013 |  |  |  |  |  |  |  |  |
| Mamo 2011 |  |  |  |  |  |  |  |  |

| **Study** | **Clear research question?** | **Data collected to answer research question?** | **Analyzing qualitative data relevant to research question?** | **Appropriate consideration given to how findings relate to context?** | **Relationship b/w researcher & participants?** | **Recruitment minimizes selection bias?**  **Measurements appropriate?** | **Groups comparable? Follow up adequate?** | **Mixed methods appropriate?** |
| --- | --- | --- | --- | --- | --- | --- | --- | --- |
| Katz 2008 |  |  |  |  |  |  |  |  |

| **Study** | **Focused issue?** | **Patients randomized?** | **Patients accounted for at conclusion?** | **Blinded?**  **Groups similar?** | **Confounding factors?** | **Results be applied to context?** | **All clinical outcomes considered?** | **Are the benefits worth the harm?** |
| --- | --- | --- | --- | --- | --- | --- | --- | --- |
| Mendis 2009 |  |  |  |  |  |  |  |  |

Table B: Quantitative Studies Quality Appraisal - Randomized Control Trials (green=yes, yellow=unknown, red=no)

Table C: Mixed Methods Quality Appraisal (green=yes, yellow=unknown, red=no)
