# Supplementary material for: A systematic review of primary care models for non-communicable disease interventions in Sub-Saharan Africa
Source: BMC Fam Pract. 2017 Mar 23;18:46. doi: 10.1186/s12875-017-0613-5 (PMC5363051; doi:10.1186/s12875-017-0613-5)
Supplement: Supplementary file 5 — A Priori themes in primary research studies for NCDs Interventions in SSA. (DOCX 18 kb) [file 12875_2017_613_MOESM5_ESM.docx]

| **Article** | **Screening**  *Case Finding* | **Prevention**  *Modify Risk Factors* | **Control: Decision Support** | | | | |
| --- | --- | --- | --- | --- | --- | --- | --- |
|  |  |  | *Standard diagnosis* | *Standard treatment* | *Referral pathway* | *Adherence* | *Follow-up* |
| Pastakia 2013 | General population (community and home-based) | Lifestyle modification counseling | SBP >160  RBS >7 mmol/L | Not clear what treatment offered at district hospital | Positive screen referred to district health clinic, secondary and tertiary referral process not described | Phone reminder of referral | HTN 31%, DM 22-23%, follow up at clinic |
| Rabkin 2012 | None | DM Peer educators | Not clear, mentions point of care diagnosis | Step-by-step protocol | Tertiary outpatient department, no referral pathway mentioned | None | Appointment system in place |
| Chamie 2012 | 5-day community based screening | Pre- and post-counseling for HTN/DM | BP > 140/90 x3, RBS >11.1 mmol/L | Not clear what offered at tertiary hospital for treatment | Local health center did not manage HTN/DM, tertiary hospital | None | HTN 43% DM 61% follow up at clinic |
| Price 2011 | None | Empowerment based diabetes group education | RBS > 12 mmol/L | Algorithm for oral hypoglycemic titration | Referred to MD for possible insulin | None | 320 patients enrolled, 80 at 4 years |
| Bloomfield 2011 | General population community and home based | Lifestyle modification counseling | SBP >160  RBS >7mmol/L | Computer based decision support with clinical algorithm | Positive screen referred to district health center | Phone reminder of referral | Aggressive outreach program traces no shows |
| Mendis 2010 | All patients between 30-70 presenting to primary health center | Counsel on diet, activity, smoking; education handout | 2 SBP’s >140 4 months apart if 50 or smoking, SBP>150 | Algorithm for titration of HCTZ for BP | BP>179 or history of myocardial infraction/ stroke/ DM/ transient ischemic attack/ positive urine for glucose referred to “next level of care” | None | 100% at 4,8,12 months for experimental arm, 75% for controls |
| Labhardt 2010 | All adult patients presenting to primary health center | Counseling on diet and lifestyle by non-physician clinician | 4 BP >140/90 for treatment  2 FBS >7 mmol/L | Yes (HCTZ +/- CCB, MF +/-GBL) | Yes if HTN or DM still not controlled refer to hospital physician | None | High attrition rate, 18% retention at 1 year |
| Kengne 2009 | Initial survey, opportunistic otherwise during routine primary health center visit | Counseling on: smoking/alcohol/ obesity/ activity/ salt intake | Average 3 BP >140/90 with DM treatment or >160/95 treatment | Clinical algorithm: HTN – HCTZ, add CCB or BB, then methyldopa or CCB/BB not used | Triple therapy for HTN not controlled referred to physician | None | Loss to follow up traced and tracked, 77% had at least 1 visit |
| Katz 2009 | Adults >18 and <80 years old screened at primary health clinic | Counseling by primary health care nurse | BP >140/90  RBS >8 | Vague algorithm, “add another class of medications for DM/HTN” | Referral if maximum therapy, serious cardiovascular comorbidity, need insulin, poor kidney function, anti-lipid meds | None | 49% of primary health care patients lost to follow up |
| Bovet 2008 | Screening of 25-64 year olds in home in 5 areas of the city | 10 minutes advice on lifestyle and nutrition | 3 BP readings at each visit, BP >165/95 on 4^th^ visit w/n 45 days | Not clear what treatment offered at local health clinic | If positive screen referred to health clinic, referral pathway beyond not defined | None | 14% lost to follow up in 45 days for diagnosis, 34% follow up in health clinic |
| Mamo 2007 | Patient with symptoms | Not clear | Doesn’t specify dx criteria | Doesn’t specify protocol used | Referral to specialist at hospital if uncontrolled | None | Didn’t record follow up of patients |
| Coleman 1998 | None | Lifestyle and dietary advice given | Persistent SBP >170 or DBP >100, DM and asthma clinically | Clinical algorithm; HTN: HTCZ, add methyldopa; DM: MF; Asthma: beta-2 agonist, steroid inhaler, oral theophylline | As per previous algorithm, if not controlled then refer to physician at district hospital | None | Doesn’t report follow up rates, 1 month follow up unless stable then 6 month follow up |

Additional file 5: *A Priori* Themes in Primary Research Studies for NCDs Interventions in SSA *(SBP = systolic blood pressure, RBS = random blood sugar, HTN = hypertension, DM = diabetes mellitus, BP = blood pressure, HTCZ = Hydrochlorothiazide, FBS = fasting blood sugar, CCB = Calcium channel blocker, MF = Metformin, GBL = Glibenclamide, BB = Beta blocker, DBP = diastolic blood pressure)*
