# Supplementary material for: A systematic review of primary care models for non-communicable disease interventions in Sub-Saharan Africa
Source: BMC Fam Pract. 2017 Mar 23;18:46. doi: 10.1186/s12875-017-0613-5 (PMC5363051; doi:10.1186/s12875-017-0613-5)
Supplement: Supplementary file 6 — A Priori themes in primary research studies for NCD interventions in SSA. (DOCX 17 kb) [file 12875_2017_613_MOESM6_ESM.docx]

| **Article** | **Control: Human Resources** | | **Control: Health Systems** | | | |
| --- | --- | --- | --- | --- | --- | --- |
|  | *Task-shifting* | *Training of staff* | *Decentralized care* | *Essential diagnostics* | *Essential medicines* | *Systematic monitoring and evaluation* |
| Pastakia 2013 | HIV counselor | 1 day training on HTN and DM | Screening – yes  District hospital – yes | BP machine  Glucometer | Not mentioned what available at hospital | Not clear how patient visits recorded at health centre |
| Rabkin 2012 | MD / RN | Ongoing training | Tertiary OPD clinic - no | BP machine, scales  Ophthalmoscope | Anti-HTN, oral hypoglycemic, insulin | Checklist / flow sheet/ sexual transmitted disease forms |
| Chamie 2012 | HIV counselor | 1 week for screening only | Tertiary hospital - no | Glucometer, BP machine | Oral hypoglycemic  Anti-HTN | Not clear how pt visits recorded at HC |
| Price 2011 | RN | British Diabetes RN trained 1 local RN | Rural PHC clinics - yes | Glucometer, scale, BP machine, urinalysis  Lab: Hba1c for study | MF, GBL | Measured Hba1c on patients at intervals |
| Bloomfield 2011 | CHW screening, MD and clinical officers | ‘Train the trainer’ approach | Screening – yes  District hospital yes | “Advanced diagnostic services for chronic NCDs” | “Advanced therapeutic services for chronic NCDS” | CVPD-specific collection of clinical data |
| Mendis 2010 | “Health care workers” | Training workshop, repeated in 2-4 month | PHC - yes | BP machine  Urinalysis - glucose | Thiazides | Data collection of risk factors, BP, medications, Past history |
| Labhardt 2010 | Non-physician clinician | 5 3-day modules, 6-monthly 1-day refresher courses | 75 PHC clinics – yes (all rural) | BP machine, glucometer  Stethoscope, Urine test strips | HCTZ, nifedipine, MF, GBL, no insulin | Standardized medical record forms |
| Kengne 2009 | RN led | 3 day course for RN’s at baseline, 6 months and 18 months | 5 PHC clinics – yes (mix of urban/rural) | BP machine, scale, glucometer, test strips, peak-flow-meters | Aminophilline, Salbutamol, MF, GBL, Gliclazide, HCTZ, Propranolol, Nifedipine, Methyldopa, Captopril, Respirine and Chlothalidone | Standardized medical record forms |
| Katz 2009 | RN led health centre | None specifically mentioned | 20 clinics – yes (mix urban/rural) | BP machine, glucometer, scale, waist circumference  Lab: ACR, lipids, hba1c | Ace Inhibitor, CCB, HCTZ, Aldomet, BB, oral hypoglycemic | Standardized medical record forms (primarily recorded for study) |
| Bovet 2008 | Clinical officers | Trained for survey method, not additional NCD | Screening – yes  Healthcare – not clear, “health care facility” | BP machine | Not clear what available at health care facility | Not clear how recorded at health care facility |
| Mamo 2007 | RN and health officer led | Continuous teaching by MDs to because staff migration | 3 rural clinics and 1 district hospital - yes | Doesn’t state | Doesn’t specify | Doesn’t specify |
| Coleman 1998 | RN led (however initial dx by MD) | No training described | Outpatient department used however 79% transferred to local clinic – yes (mostly) | BP machine, stethoscope, no glucometer/peak flow meter | HCTZ, methyldopa | Medical charting, not standardized |

Additional file 6: *A Priori* Themes in Primary Research Studies for NCD Interventions in SSA *(HIV = Human immunodeficiency virus, HTN = Hypertension, DM = Diabetes mellitus, BP = Blood pressure, MD = Medical doctor, RN = Registered nurse, MF = Metformin, GBL = Glibenclamide, HbA1C = Hemoglobin A1C, NCD = Non-communicable disease, CHW = Community health worker, CVPD = Cardiovascular pulmonary disease, HCTZ = Hydrochlorothiazide, ACR = Albumin creatinine ratio, CCB = Calcium channel blocker, BB = Beta blocker*
